# Does power distance in healthcare teams linked to patient satisfaction? A multilevel study of interprofessional care teams in a referral hospital in Indonesia

**DOI:** 10.1186/s12913-023-10534-3

**Published:** 2024-01-16

**Authors:** Susi Afrianti Rahayu, Sunu Widianto, Irma Ruslina Defi, Rizky Abdulah

**Affiliations:** 1 Department of Pharmacology and Clinical Pharmacy, Faculty of Pharmacy, Universitas Padjadjaran, Jatinangor, 45363 Indonesia; 2 Bumi Siliwangi College of Pharmacy, Bandung, Indonesia; 3 Department of Management and Business, Faculty of Economics and Business, Universitas Padjadjaran, Jatinangor, 45363 Indonesia; 4 Department of Physical Medicine and Rehabilitation, Faculty of Medicine, Universitas Padjadjaran, Jatinangor, 45363 Indonesia; 5 Department of Physical Medicine and Rehabilitation, Hasan Sadikin Hospital, Bandung, Indonesia; 6 Center for Excellence for Pharmaceutical Care Innovation, Universitas Padjadjaran, Jatinangor, Indonesia

**Keywords:** Hospital, Interprofessional care team, Power distance, Patient satisfaction

## Abstract

**Background:**

Interprofessional care teams collaborate to provide care to patients in hospitals to ensure their full recovery. To provide quality patient care, healthcare workers must have a comprehensive understanding of each other’s roles and collaborate effectively. Good interpersonal skills are also essential for maintaining cooperative and collaborative relationships, listening, and respecting other team member’s values and positions. Therefore, this study aimed to investigate the effect of power distance in interprofessional care on patients’ satisfaction.

**Method:**

A quantitative study was conducted in a hospital by using a questionnaire instrument to collect information from patients and members of the interprofessional care team. The respondents included 10 geriatric, 19 palliative, 36 cancer, 8 burn, and 18 medical intermediate care (MIC) teams. Subsequently, a hierarchical regression analysis was conducted to examine whether interprofessional care could significantly predict the relationship between team power distance and patient satisfaction.

**Results:**

The measurement of the effect of power distance in interprofessional care among doctors, nurses, pharmacists, and nutritionists on patient satisfaction revealed nonsignificant results. However, the final analysis indicated negative coefficients with regard to power distance for nutritionists (-0.033098), nurses (-0.064912), and pharmacists (-0.006056). These findings indicated that the power distance associated with these professions was linked with decreased patient satisfaction.

**Conclusions:**

The results suggested that power distance within an interprofessional care team can reduce patient satisfaction.

## Introduction

Hospitals are considered to be complex and intricate organizations due to the fact that they require members of various health and nonhealth professions to work together to improve patients’ health. To achieve this goal, collaboration among professionals is necessary, and many hospitals have established interprofessional care teams. Health workers must also understand each other’s roles and work together effectively to provide quality care for patients. Members of the interprofessional care team must have good interpersonal skills to maintain their relationships in terms of cooperating, collaborating, listening, and respecting each other’s values or positions.

The performance of an interprofessional care team can influence patient satisfaction [1]. Patient satisfaction refers to patients’ reactions to the experiences they have received [2]. Interaction and communication within an interprofessional care team have positive impacts on patient and family satisfaction [3]. Interprofessional care teams, which contain members of various professions, exhibit gaps in power distance, which can be observed in terms of members’ educational backgrounds. Such gaps may lead to patient dissatisfaction with the services provided. A significant level of power distance is evident in interprofessional care teams, ultimately influencing the services they provide and, consequently, impacting patient satisfaction.

Interprofessional care teams in hospitals are dominated by members of the medical profession due to their extensive medical knowledge of health care. One study on power distance showed that doctors feel more confident when they act on pharmacists’ recommendations regarding drug management. Moreover, to reduce power distance, pharmacists must perform their jobs effectively, thus emphasizing the importance of professional commitment [4].

According to one report, diversity within a team is associated with increased value and benefits [5] and can influence group performance by increasing or even decreasing productivity and performance satisfaction. This report indicated that diversity can be a double-edged sword with regard to performance [6].

The multiprofessional services provided by hospitals have the potential to cause issues pertaining to overlapping services, interprofessional conflicts, and delays in examinations and actions. Approximately 70–80% of errors in health services are caused by poor communication and understanding within the care team. Although effective teamwork has been found to reduce patients’ safety problems [7], the majority of patients appear to be unconcerned about the performance of interprofessional healthcare teams [8].

Individual roles and team identity are important concepts in the context of interprofessional collaboration. One obstacle to the realization of this concept is the difficulty of achieving a sense of belonging within a team due to differences in culture, processes and formal communication [9]. Hitherto, no studies have investigated the effect of power distance in interprofessional healthcare on patient satisfaction; therefore, this research addresses this gap by conducting a multilevel study of interprofessional care teams. In this study, we investigate how power distance in the context of collaborative practices involving patients can affect interprofessional care and patient satisfaction.

## Method

This study was conducted at a referral hospital in Bandung City, Indonesia. The interprofessional care teams selected for this study featured a complete set of members, including doctors, nurses, pharmacists, and nutritionists, all of whom provided patient care. A total of 10 geriatric, 19 palliative, 36 cancer, 8 burn, and 18 medical intermediate care (MIC) teams participated in the study.

The inclusion criteria for respondents focused on hospital employees, leaders and members of the aforementioned teams, patients or family members of inpatients who were treated by one of these teams, and the ability to communicate to ensure that respondents could complete the questionnaire effectively. The exclusion criteria focused on respondents younger than 17 years old and individuals who objected to completing the questionnaire.

Primary data were collected by performing a cross-sectional study that involved three questionnaires adapted from previous research, i.e., one questionnaire each regarding power distance [10], interprofessional care [11], and patient satisfaction [12].

The questionnaire was subjected to a process of translation and back-translation performed by independent professional translators. A pilot study was conducted by reference to 30 respondents to test the validity and reliability of the questionnaire. The Indonesian version of the questionnaire was shown to be valid, with the correlation values for each question with regard to the total score being > 0.7 [13]; this version of the questionnaire was also found to be reliable, with Cronbach’s alpha coefficients of 0.875, 0.875, and 0.952 for power distance, interprofessional care, and patient satisfaction, respectively. This process resulted in a final questionnaire featuring 26 questions (Table 1).

The power distance questionnaire was operationalized based on several indicators. The first such indicator was the ability and responsibility of the leader with regard to making his or her own decisions, which was covered by questions 1, 2, and 4. The second indicator focused on the inability of the leader of the interprofessional care team to express disagreement; this item was covered by question 3. The third indicator was the perception that the leader would lose power if subordinates were involved in making decisions; this indicator was covered by question 5.

Interprofessional care was operationalized using several indicators. The first such indicator focused on the influences on patient clinical outcomes and was covered by questions 1 and 2. The second indicator was focused on the influences on coordination among professions, which was covered by question 3. The third indicator was the role of pharmacists in interprofessional care in the context of drug administration therapy, which was covered by question 4.

Patient satisfaction was also operationalized using several indicators. The first such indicator focused on the patient’s experience with the healthcare service he or she received, which was covered by questions 1, 2, 3, 4, 5, and 6. The second indicator was the patient’s experience with the performance of health workers, which was covered by questions 7, 8, 9, 10, and 11. The third indicator was the patient’s belief in the success of the therapy provided, which was covered by questions 12, 13, and 14. The fourth indicator was the patient’s overall satisfaction with the hospital, which was covered by questions 15, 16, and 17.

The sample was recruited using a nonprobability sampling method, namely, purposive sampling, and consisted of 91 interprofessional care teams. The independent and dependent variables were team power distance and patient satisfaction, respectively, while the mediator was interprofessional care. The data were obtained using a survey method that involved distributing questionnaires that had been tested with regard to validity and reliability.

### Ethical considerations

All methods were implemented in accordance with the relevant guidelines and regulations. This study was approved by the Research Ethics Committee of Universitas Padjadjaran No: 566/UN6.KEP/EC/2020. In addition, we confirmed that informed consent was obtained from all the subjects and/or their legal guardian(s). The security of the data was ensured, and the data could be accessed by the author only after the process of anonymous questionnaire completion.

### Analysis design

This study conducted a hierarchical regression analysis to investigate whether interprofessional care significantly could predict the correlation between team power distance and patient satisfaction.

The measurement of the power distance variable focused on the team, and the variables emphasized the team level. Individuals on each work team were asked to complete a questionnaire based on their perceptions and assessments of power distance at work. The measurement of interprofessional care and patient satisfaction variables focused on the individual level.

The magnitude of the effect was measured after treatment using effect size measurements. Effect size is generally used in studies featuring large populations and variables [14]. Interpretation of the effect size results was performed according to Cohen’s rules: small (0 < d ≤ 0.2), moderate (0.2 < d ≤ 0.5), large (0.5 < d ≤ 0.8), or very large (> 0.8).

### Models and analysis

#### Hierarchical linear modeling (HLM)

**Fig. 1:**
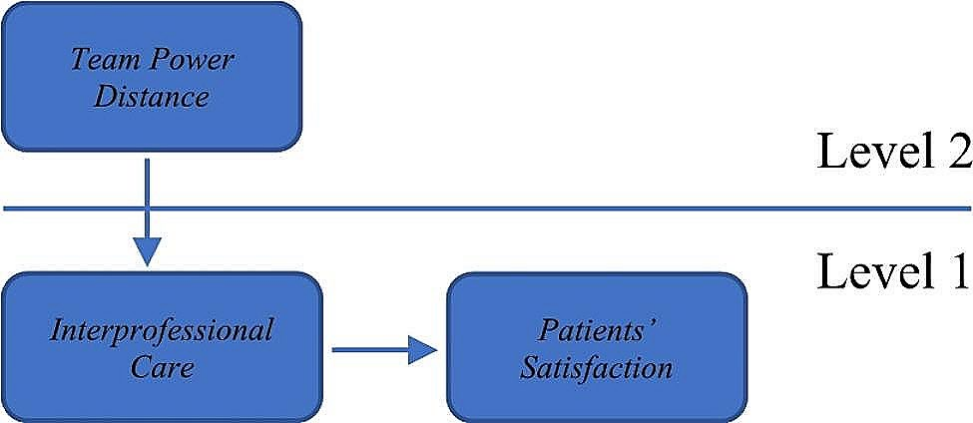
Study model

Figure 1 shows that in this study, team power distance was set at level 2, while interprofessional care and patient satisfaction were set at level 1. A 2-1-1 model presented the unit of analysis at level 2 as an independent variable (team power distance), the unit of analysis at level 1 as a mediating variable (interprofessional care), and the unit of analysis at level 1 as the dependent variable (patient satisfaction).

## Results

A total of 91 interprofessional care teams, each of which included doctors, nurses, pharmacists, and nutritionists, participated in this study, and 455 questionnaires were successfully collected from the respondents, who were healthcare professionals. Moreover, 89 patients, including 63 females and 28 males, also participated in the study. In terms of education level, a plurality of respondents were high school graduates (49%), and the most common age range was 31–40 years (34%).

Table 1 presents team power distance, interprofessional care, and patient satisfaction as the independent, mediating, and dependent variables included in this research. The items in the survey were scored on a 5-point Likert-type scale ranging from 1 to 5, where 1 represents strongly disagree, 2 represents disagree, 3 represents undecided, 4 represents agree, and 5 represents strongly agree. The reliability of each variable was calculated using Cronbach’s alpha coefficients.

**Table 1:** Validity and reliability test results

No	Statements	Correlation coefficient	R table
	Power distance Cronbach’s alpha = 0.875		
1	In many situations, a leader must make decisions without consulting subordinates	0.421	0.367
2	When a leader makes decisions, an employee should not question them	0.763	0.367
3	A leader must not express disagreement with an employee	0.801	0.367
4	A leader makes the correct decisions without consulting others	0.589	0.367
5	A leader who involves an employee in decisions will lose power	0.597	0.367
	Interprofessional care Cronbach’s alpha = 0.875		
1	Interprofessional care in my practice results in improved patients’ outcomes	0.765	0.367
2	Interprofessional care in my practice results in increased clinical efficiency	0.732	0.367
3	Interprofessional care in my practice results in improved coordination of care and patient follow-up	0.869	0.367
4	Drug use reviews presented by pharmacists in my practice result in fewer drug-related problems as part of interprofessional care	0.737	0.367
	Patients’ satisfaction Cronbach’s alpha = 0.952		
1	Health workers are very fast with regard to receiving and responding to phone calls	0.666	0.553
2	The registration procedure ensures a convenient consultation	0.823	0.553
3	Sufficient information about the waiting time is provided beforehand	0.721	0.553
4	The waiting time for examination and treatment is acceptable	0.736	0.553
5	The payment process is convenient	0.926	0.553
6	Payment items on the receipt are easy to understand	0.926	0.553
7	The appearance of the health team is very neat	0.624	0.553
8	The health team is kind and polite	0.856	0.553
9	Information about treatment is always given by health workers beforehand	0.856	0.553
10	The health team pays attention to my conversation (question)	0.939	0.553
11	The health team provides an adequate explanation of the symptoms and treatment plan, so it is easy to understand	0.864	0.553
12	Effective remedies	0.856	0.553
13	Reliable treatment	0.763	0.553
14	Medications and prescriptions are appropriate	0.939	0.553
15	I am satisfied with this hospital overall	0.874	0.553
16	I intend to continue using this hospital	0.787	0.553
17	I will recommend this hospital to others	0.790	0.553

Table 2 shows the parameter estimation, standard error estimation, and P value for each analysis with regard to the HLM. The results showed that the health professions represented in the interprofessional care team had little influence on patient satisfaction, and the power distance associated with relationships within the interprofessional care team were not associated with any significant results. With respect to this parameter, the scores for patient satisfaction with the interprofessional care provided by the doctor, nutritionist, nurse, or pharmacist were 0.629, 0.533, 0.279, and 0.908, respectively, while those pertaining to power distance were 0.073, 0.003, 0.028, and 0.007, respectively. Moreover, the negative coefficients pertaining to power distance with regard to nutritionists, nurses, and pharmacists were -0.033098, -0.064912, and -0.006056, respectively. These findings indicated that power distance among professions reduced patients’ satisfaction.

**Table 2:** HLM data processing results

	Coefficient	Standard error	P value
**Doctor**			
For intrcpt 1, β_0_			
Intrcpt 2, γ_01_	4.436264	0.042348	< 0.001
Powerdis, γ_01_	0.026318	0.054247	0.629
For the icaredoctor slope, β_1_			
Intrcpt 2, γ_10_	0.164197	0.090642	0.073
**Nutrition**			
For intrcpt 1, β_0_			
Intrcpt 2, γ_01_	4.436264	0.041571	< 0.001
Powerdis, γ_01_	-0.033098	0.052900	0.533
For the icarenutritition slope, β_1_			
Intrcpt 2, γ_10_	0.212567	0.069044	0.003
**Nurse**			
For intrcpt 1, β_0_			
Intrcpt 2, γ_01_	4.436264	0.042127	< 0.001
Powerdis, γ_01_	-0.064912	0.059612	0.279
For the icarenurse slope, β_1_			
Intrcpt 2, γ_10_	-0.217294	0.097204	0.028
**Pharmacist**			
For intrcpt 1,β_0_			
Intrcpt 2, γ_01_	4.436264	0.041571	< 0.001
Powerdis, γ_01_	-0.006056	0.052132	0.908
For the icarepharmacist slope, β_1_			
Intrcpt 2, γ_10_	0.261367	0.094249	0.007

The multivariate test results (Table 3) indicate a very small significance value of 0.0092 for the effect size measurement; thus, the difference in the influence of power distance in interprofessional care on patient satisfaction represented only a very small effect, and it was also associated with a small effect size.

**Table 3:** Differences in the influence of power distance in interprofessional care on patient satisfaction

Effect	Wilks Lambda	F	Hypothesis df	Sig	Effect size (d)
Interprofessional care	0.0947	2.45	2	0.092	0.053

## Discussion

A total of 91 interprofessional healthcare teams were involved in this study. Our study showed that all four types of health professionals included in the interprofessional care team contributed to patient satisfaction. The existence of power distance within the interprofessional care team caused differences in the significance of each health profession. The results revealed that no profession had a significant effect on patient satisfaction, in which context pharmacists had the least effect. This finding can be attributed to the fact that clinical pharmacists in hospitals have only limited interactions with patients. To ensure the success of an interprofessional care team, the recommended strategies include building trust among multiple professionals in terms of circles of quality, encouraging government and health authorities to promote unity among multiple health professionals, and encouraging pharmacists to be more proactive [15].

A total of 77.9% of the respondents agreed that doctors are responsible for final decisions regarding patient care. This finding revealed that the collaborative practice model in Indonesia was similar to traditional or hierarchical collaborative practice [16]. In primary care, general practitioners across Europe, the US, and Australia are supported by other qualified healthcare professionals, including pharmacists. Although it did not focus on the same level, this approach has yielded positive results in terms of the clinical development of patients [17].

This study revealed that the power distance associated with doctors, nurses, nutritionists and pharmacists exhibited negative correlations with patient satisfaction, indicating that the existence of power distance can reduce patient satisfaction. Previous investigations have established that doctors are the main persons responsible for interprofessional care collaboration when dealing with patients. However, doctors cannot work alone because other health workers are also needed for providing care to patients. Health workers are always expected to collaborate and work together to improve patients’ health. The emergence of disputes or disagreements among health professionals when providing treatment can be caused by mismatches among health workers in terms of competence, poor teamwork skills, and weak leadership roles [18].

The quality of interprofessional teamwork is supported by the quality of the interactions among various professions. The form of hierarchy observed in interprofessional teams often involves the professional acting as a leader, and the factors that can influence this hierarchy include seniority, experience, and culture [19]. The dominance of a doctor can be notable within the existing hierarchical social culture [20]. The events observed in this research also explain why doctors have a great deal of control with regard to patient treatment. The results reveal a positive coefficient (0.026) of power distance for doctors, thus indicating that power distance in the medical profession does not reduce patient satisfaction as in other professions. According to the Ministry of Health (2022), the leader of an interprofessional care team is a responsible doctor [21]. However, the imbalance among professionals in terms of hierarchy and power as well as the existing lack of understanding of professional competence are challenges that must be overcome in the context of interprofessional care collaboration [22].

For health workers on interprofessional care teams in hospitals, collaboration can increase job satisfaction and retention. Team tasks can be more predictable, less urgent, and less complex when health workers who provide collaborative patient care are well coordinated [23]. Moreover, effective collaboration requires mutual accountability among individuals, including a clear division of tasks and roles. This situation is also related to team concepts such as perceptions of psychological security and power distance [24], which can shape interactions within interprofessional care teams [25]. However, a hierarchical social culture featuring a wide power distance in the global community represents a significant challenge for interprofessional care [26].

The results of this research indicated that the power distance among the four types of health workers in interprofessional care teams had only limited statistical value (> 0.001). Power distance was found to be negatively related to the relationship between doctors and nurses [27]. With regard to the pharmacist role, the presence of power distance on the interprofessional care team can reduce the effectiveness of collaboration. A previous study conducted in China reported that power distance influences the relationship between safety emphasis and the fear of reporting medication errors exhibited by nurses [28]. The exchange of information regarding treatment can be hindered by power distance, in which context communication is hierarchically based, and the corresponding dynamics can determine what information is acceptable within a team [29]. Events in the hospital show that communication within the interprofessional care team occurs mostly in a nonverbal form, in which context the relevant actors communicate based on the information contained in the medical records at hand. Collaborative care involves patients playing a role in treatment selection [30], while the interprofessional care team supports patients both technically and emotionally with regard to making treatment decisions [31]. The close relationships among health professions, good communication and attitudes, effective and efficient personnel, and the belief of each profession in the importance of collaboration are factors that affect the quality of collaboration, while the existence of hierarchy and power distance can hinder collaboration [32].

Doctors’ maintenance of a degree of power distance from other professions by limiting their interprofessional relationships hinders communication in the context of collaboration [33]. Namely, communication must be established in the context of a partnership; therefore, doctors who become team leaders in interprofessional care should operate as organizational machines who are responsible for organizing and coordinating interprofessional care teams and guiding teams to develop regularly [34]. A common understanding among healthcare professionals should be introduced early in interprofessional education, as it is critical for preparing students to enter their future work environment. A previous study suggested that as the power distance between medical students and nursing students decreases, the psychological safety of the entire interprofessional care team increases, thus indicating that power distance impacts team effectiveness [24].

The results of this study also showed that the influence of power distance in interprofessional care on patient satisfaction is small. This finding is in line with a previous report that reported that communication can still lead to the creation of a partnership [35]. A clear agreement regarding patients, responsibilities, and communication is necessary for the successful provision of interprofessional care [36]. Communication between nurses and doctors was found to be crucial with regard to whether nurses can provide the best quality care in collaboration with doctors [37]. The existence of power distance between nurses and doctors was associated with a significant negative correlation. Power distance refers to the degree to which weak members of an organization can expect power to be distributed unequally and accept this situation [26]. Partnerships in interprofessional care strengthen the roles of team members. Pharmacists on an interprofessional care team perform their roles well when they are supported by other professionals on the team [38]. Furthermore, doctors, nurses, pharmacists, and nutritionists can have a positive influence on patients’ care by aligning and strengthening collaboration [39].

Nutritionists responded to existing power relationships by building and maintaining relationships, advocating for patients, and negotiating decisions with other healthcare staff with the goal of improving nutritional outcomes and patient outputs as well as with the aim of enhancing communication skills within interprofessional care teams [40]. The level of collaboration was associated with varying results in healthcare settings, in which context the interaction between nutritionists and other members of the interprofessional care team was the least impactful due to their overlapping scope of practice as well as the existence of limitations in shared practice spaces [41].

Good knowledge and skills in interprofessional care were very effective with regard to meeting patients’ satisfaction and expectations [42] and addressing patients’ complex needs [43]. Patients with chronic conditions have positive experiences when they are treated by a health collaboration team [44]. However, high knowledge and skill gaps can lead to low levels of respect within interprofessional care teams [45]. Therefore, sharing knowledge and skills in the context of collaborative interactions within interprofessional care teams is necessary for increasing the quality of patient care [46]. Patients’ information sharing should also be prioritized in interprofessional care communication [47]. Since treatment is provided in accordance with patient needs, the mode of administration affects the clinical outcomes of patients [48]. The delivery of quality health services can meet the needs of patients more effectively [49]. The existence of power distance is one of the causes of the emergence of the historically hierarchical form of health services based on the professions of the relevant actors [50]. The provision of suboptimal health services has a negative impact in this context. The types of activities usually performed by interprofessional care teams are in line with the particular competencies of each team member. Healthcare professionals perform duties in accordance with their competence; for example, doctors perform patient diagnoses and other clinical activities; nurses engage in professional nursing practices and practices based on ethics, law and cultural sensitivity; and nutritionists develop food standards that patients are supposed to follow. Pharmacists are responsible for assisting patients in the provision of treatment, especially with regard to the administration of medicines.

### Limitations

However, our study has several limitations. The data used were obtained from only one hospital; therefore, it is necessary to collect data from different teams and hospitals in the future to investigate the roles of interprofessional care relationships in healthcare in further detail. Furthermore, the interprofessional care team on which this study focused consisted of only four professions, and the inclusion of other healthcare professionals could be beneficial to improve our understanding of this issue further.

## Conclusion

This study revealed that the negative coefficients of the power distance associated with nutritionists, nurses, and pharmacists were -0.033098, -0.064912, and -0.006056, respectively. This finding indicated that power distance associated with these professions reduced patients’ satisfaction.

## Data Availability

All data generated or analyzed during this study are included in this published article and its supplementary information files, as well as further information, are available from the corresponding authors on reasonable request.

## Declarations

### Authors’ information

SAF is a PhD candidate at the Department of Pharmacology and Clinical Pharmacy, Faculty of Pharmacy, Universitas Padjadjaran, Jatinangor, Indonesia. She is also a lecturer at Bumi Siliwangi College of Pharmacy, Bandung, Indonesia. SW is an Associate Professor at the Department of Management and Business, Faculty of Economics and Business, Universitas Padjadjaran, Jatinangor, Indonesia. IRD is an Associate Professor at the Department of Physical Medicine and Rehabilitation, Faculty of Medicine, Universitas Padjadjaran, Jatinangor, Indonesia. She is also the Head of Geriatric Rehabilitation Division at Hasan Sadikin Hospital, Bandung, Indonesia. RA is a Professor at the Department of Pharmacology and Clinical Pharmacy, Faculty of Pharmacy, Universitas Padjadjaran, Jatinangor, Indonesia.

## Abbreviations

MIC: Medical Intermediate Care
HLM: Hierarchical Linear Modeling
BPPDN: Beasiswa Pendidikan Pascasarjana Dalam Negeri (Domestic Postgraduate Education Scholarship)
